# ADP-riboxanation: a new pyroptosis evasion strategy

**DOI:** 10.1093/jmcb/mjab077

**Published:** 2021-12-09

**Authors:** Heyu Li, Fangfang Zhou, Long Zhang

**Affiliations:** MOE Laboratory of Biosystems Homeostasis and Protection and Innovation Center for Cell Signaling Network, Life Sciences Institute, Zhejiang University, Hangzhou 310058, China; School of Medicine, Zhejiang University City College, Hangzhou 310015, China; Institutes of Biology and Medical Science, Soochow University, Suzhou 215123, China; MOE Laboratory of Biosystems Homeostasis and Protection and Innovation Center for Cell Signaling Network, Life Sciences Institute, Zhejiang University, Hangzhou 310058, China

**Figure 1:**
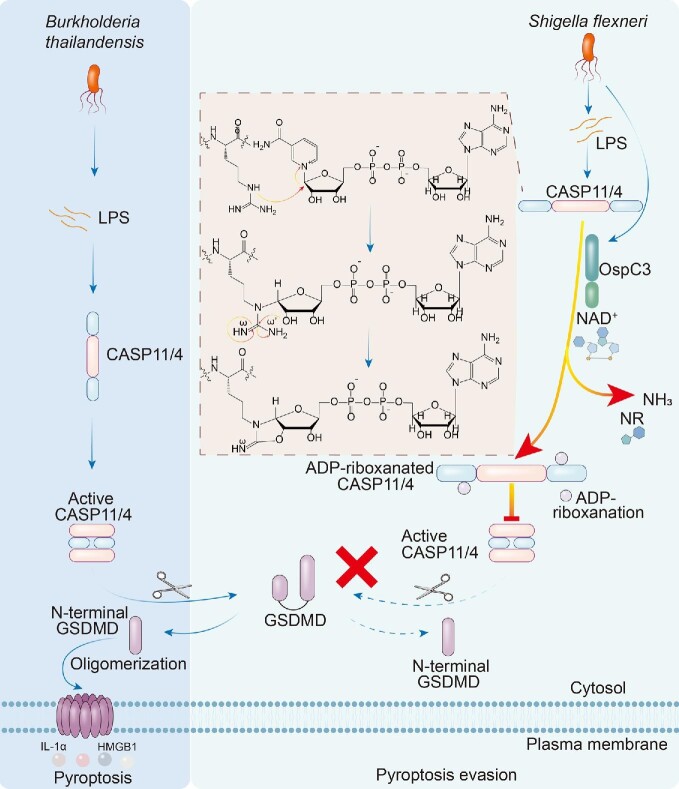
Pyroptosis evasion strategy of *S. flexneri*. OspC3 suppresses the proteolytic activity of caspase-11/4-p20/p10 by catalyzing ADP-riboxanation on Arg314 and Arg310 in caspase-4 and caspase-11, respectively. First, the arginine residue of caspase-11/4 undergoes ADP-ribosylation, after which Nam derived from NAD^+^ is released. Next, N^ω^ of the arginine side chain deaminates with the hydroxyl of ADP, generating a new modification: ADP-riboxanation. Modified caspase-11/4 fails to autoactivate and to cleave GSDMD, leading to the blockade of LPS-induced pyroptosis.

A recent study published in *Nature* by Li et al. (2021) revealed the underlying mechanism by which *Shigella flexneri* evades host pyroptosis using a type III secretion system (T3SS) effector, OspC3. OspC3 suppresses the proteolytic activity of caspase-11 or caspase-4 (hereafter referred to as ‘caspase-11/4’) in a nicotinamide adenine dinucleotide (NAD^+^)-dependent manner by catalyzing adenosine diphosphate riboxanation (ADP-riboxanation) on arginine 314 (Arg314) and Arg310 in caspase-4 and caspase-11, respectively (Figure 1). In addition, *S. flexneri* Δ*icsA*Δ*ospC3* is a live-attenuated vaccine candidate.

Pyroptosis is a programmed cell death pathway executed by the gasdermin family. The main types of activation pathways (canonical and noncanonical) are mediated by caspase-1 or caspase-4/5/8/11. During primary bacterial infection, bacterial lipopolysaccharide (LPS) triggers autoproteolytic activation of caspase-4/5/11; active caspase-4/5/11 subsequently cleaves gasdermin D (GSDMD), stimulating the host immune defense (Kayagaki et al., 2015; Shi et al., 2015). Since pyroptosis plays a vital role during infection, multiple inhibitors have been identified to block pyroptosis using various mechanisms at different steps, similar to pathogens. A large number of pathogens have evolved several ways to escape or counteract pyroptosis through unknown mechanisms, making this an indeterminate research area (LaRock and Cookson, 2012).

*Shigella* is a genus of cytosol-invading, gram-negative bacteria, which is genetically closely related to *Escherichia coli. Shigella* causes substantial morbidity and mortality with symptoms of severe diarrhea, fever, and stomach cramps, especially in young children (Zychlinsky et al., 1992). However, unlike *E. coli-*activated pyroptosis (Shi et al., 2014), *Shigella* has evolved to escape from the immune system without triggering noncanonical pyroptosis. It is reported that OspC3, a T3SS-mediated effector, interacts with the caspase-4-p20 subunit and thus prevents the heterodimerization of caspase-4-p20 and caspase-4-p10 to restrain epithelial cell death (Kobayashi et al., 2013). However, the mechanisms in the *Shigella* infection process are still unknown.

Li et al. (2021) first confirmed that *S. flexneri* evades caspase-11/4-mediated pyroptosis and identified OspC3 as the main effector against pyroptosis. In contrast to *Burkholderia thailandensis* and *Salmonella Typhimurium* Δ*sifA, S. flexneri* caused similar mortality rates in wild-type (WT) and *Casp11*^–/–^ mice. Furthermore, GSDMD cleavage was interfered with by *S. flexneri* infection in immortalized bone marrow-derived macrophages. Interestingly, *S. flexneri* Δ*ospC3* infection increased N-terminal GSDMD release and SiHa cell death. This effect was reversed upon introduction of pOspC3. The results prove that *S. flexneri* inhibits noncanonical pyroptosis through OspC3.

Second, the authors investigated the OspC3-mediated pyroptosis suppression mechanism. WT, *CASP4*^−/−^, or OspC3-expressing SiHa cells were infected with *S. flexneri* Δ*ospC3, S. Typhimurium* Δ*sifA*, and LPS, respectively. No significant differences in cell death rates were observed among groups, indicating that OspC3 played a bacteria-independent role. The authors then performed co-immunoprecipitation to detect the interaction between OspC3 and caspase-4. The binding of OspC3 to caspase-4 was found without triggering p20–p10 dissociation, contrary to a previous report (Kobayashi et al., 2013). Furthermore, OspC3 did not influence caspase-11/4 autoprocessing *in vitro*. These results show that OspC3 blocks pyroptosis through a cell-dependent but bacteria-independent mechanism.

Surprisingly, the authors found that caspase-4-p10 from 293T cells migrated slower in sodium dodecyl sulfate gel in the presence of OspC3 than it alone. In addition, the caspase-11/4-p30-C/A protein band showed a downshift in the native gel when coexpressed with OspC3 in *E. coli*. These results indicate that OspC3 promotes posttranslational modification of caspase-11/4 *in vivo*.

Next, the authors expressed caspase-11/4-p30-C/A either alone or with OspC3 in bacteria to elucidate this modification. They performed electrospray ionization–mass spectrometry (ESI‒MS) and collision-induced dissociation‒MS on purified caspase-11/4 to identify the mass alteration and the modified group. A 524-Da mass shift and adenine, adenosine monophosphate (AMP), and ADP mass fragments were found with modified caspase-11/4 by mass spectrometry, indicating that the mass shift could be attributed to adenine, AMP, and ADP in an OspC3-dependent manner. Furthermore, the *in vitro* reaction revealed that the modification of caspase-11/4-p30-C/A depended on NAD^+^. Based on the analysis, the authors took note of ADP-ribosylation (Lüscher et al., 2018), which leads to a 541-Da shift. Then, a sequence of procedures was carried out to determine why caspase-11/4 underwent a 524-Da mass shift modification, 17 Da smaller than ADP-ribosylation.

First, the authors identified the modified sites (Arg314 and Arg310 in caspase-4 and caspase-11, respectively) using ESI‒MS and considering the 524-Da mass shift as a clue. Furthermore, in caspase-4 modification reactions, increased nicotinamide (Nam) and decreased NAD^+^ levels were detected using high-performance liquid chromatography–MS quantification. Upon further calculation, the authors assumed that one Nam molecule was released as one NAD^+^ molecule was incorporated into caspase-11/4, indicating ADP-ribosylation (Lüscher et al., 2018). Subsequently, to identify the molecule for which the 17-Da mass shift occurred, they used different NAD^+^ analogues in the *in vitro* assay. They confirmed Nam as the leaving group and a 17-Da loss on the phosphoribosylated arginine using stable isotopic labelling for amino acids. Furthermore, by incorporating 2′-H-NAD^+^, 2′-F-NAD^+^, and ninhydrin, the authors clarified that the arginine N^δ^ was the atom where the initial ADP-ribosylation occurred and the arginine N^ω^ underwent deamination. Overall, the authors elucidated two steps for this modification and named it ADP-riboxanation.

The ADP-riboxanation catalytic activity of OspC3 raised the question of whether other members of the OspC3 family have similar functions. By analyzing the domain and the vital residues of ADP-riboxanase, the authors asserted that the ARD domain of OspC3 determines caspase-11/4 recognition, whereas its N-terminal domain functions as the ADP-riboxanase. In addition, the authors proved that Arg314 ADP-riboxanation in caspase-4 is critical in pyroptosis inhibition by OspC3.

Finally, to test the impact of ADP-riboxanation on *S. flexneri* infection, the authors generated OspC3 ADP-riboxanase-dead E192A/H328A mutant (EH/AA) *S. flexneri*. Lower bacterial loads were observed in mice infected with *S. flexneri* Δ*ospC3* overexpressing EH/AA OspC3 than in those infected with WT OspC3. Furthermore, mice generated more anti-*Shigella* IgG when infected with *S. flexneri* Δ*ospC3.* Consequently, these mice were more resistant to the lethal *S. flexneri* re-infection, indicating that the *S. flexneri* Δ*ospC3* strain could serve as a promising candidate for a *Shigella* vaccine.

Li et al. (2021) first clarified that *S. flexneri* reduced pyroptosis via OspC3, an ADP-riboxanation covalent enzyme on caspase-11/4, which is a new pyroptosis hijacking strategy (LaRock and Cookson, 2012). Modified caspase-11/4 failed to undergo autoprocessing and inhibited pyroptosis. This study expands our knowledge on how bacteria interfere with LPS-induced pyroptosis. More interestingly, the ADP-ribosylation of caspase-11/4 modified by D117A-OspC3 can be erased by ADP-ribosylhydrolases, while ADP-riboxanation remains stable, which indicates the unique role of ADP-riboxanation in *Shigella* infection. There are many barriers impeding the development of the *Shigella* vaccine (Barry et al., 2013). The current study on *Shigella* pathogenesis identifies an important virulence factor, OspC3. *S. flexneri* Δ*icsA*Δ*ospC3* induced a higher level of anti-*Shigella* IgG compared with *S. flexneri* Δ*icsA*, the live-attenuated vaccine currently available. Hence, *S. flexneri* Δ*icsA*Δ*ospC3* could serve as potential vaccine candidate.

There are still several questions to be answered in future research. For example, structural biology could elucidate the advantages of ADP-riboxanation compared with ADP-ribosylation and why arginine-ADP-riboxanated caspase-11/4 loses its proteolytic activity (Wang et al., 2020). Exploring ways to restrain the function of ADP-riboxanated arginine may help develop effective medications against *Shigella* with minor side effects. In addition, it is worth investigating whether ADP-riboxanation and arginine deamination exist widely in eukaryotes to reveal the significance of ADP-riboxanation in evolution. It is also of great value to investigate whether ADP-riboxanation exists in other amino acids with special side chains, such as histidine and glutamine. It would be interesting to exploit the formation of the oxazolidine ring during other posttranslational modification processes.

In conclusion, the authors present a new perspective on bacterial anti-pyroptosis and present a promising *Shigella* vaccine candidate. In future studies, it will be important to reveal other virulence factors that could serve as potential vaccine antigens or as targets for treatment together with chemical compound screening.

*[This work was supported by a special program from the Ministry of Science and Technology of China (2016YFA0502500 to L.Z.), the National Natural Science Foundation of China (U20A201376, 31925013, 3212500161, 82041009, 31871405, 31701234, 81902947, 82041009, 31671457, 31571460, and 91753139), Jiangsu Provincial Distinguished Young Scholars award (BK20180043), the Key Project of University Natural Science Foundation of Jiangsu Province (19KJA550003), and a project funded by the Priority Academic Program Development of Jiangsu Higher Education Institutions and the Postgraduate Research & Practice Innovation Program of Jiangsu Province (KYCX17_2036).]*
